# Music Makes the World Go Round: The Impact of Musical Training on Non-musical Cognitive Functions—A Review

**DOI:** 10.3389/fpsyg.2015.02023

**Published:** 2016-01-07

**Authors:** Sarah Benz, Roberta Sellaro, Bernhard Hommel, Lorenza S. Colzato

**Affiliations:** ^1^Institute of Experimental Psychology, Heinrich-Heine UniversityDüsseldorf, Germany; ^2^Cognitive Psychology Unit and Leiden Institute for Brain and Cognition, Leiden UniversityLeiden, Netherlands

**Keywords:** musical training, cognitive training, neuroplasticity, executive functions, memory, processing speed, creativity, attention

## Abstract

Musical training is becoming increasingly popular as a topic for scientific research. Here we review the available studies investigating whether and to which degree musical experience generalizes to cognitive functions unrelated to music abilities in healthy humans. In general, it seems that musical training is associated with enhancing effects, even if sometimes only restricted to the auditory domain, on various cognitive functions spanning from executive control to creativity. We conclude that musical engagement may be a useful cognitive training to promote cognitive enhancement, but more research using longitudinal studies and taking into account individual differences is necessary to determine actual benefits.

## Introduction

The interest in the influence of musical experience on our daily life is constantly growing. In the past decade, this has motivated various studies on the possible cognitive benefits that having learned to play musical instruments may have on the players. Not surprisingly, musicians have been reported to be better than non-musicians in various music-related skills (see, Schellenberg, 2005; Zatorre et al., 2007, for reviews on the topic), which, however, mainly demonstrate the efficiency of the training program. Interestingly, however, similarly to other fields such as bilingualism (Bialystok et al., 2012), video gaming (Bavelier et al., 2012), and meditation (Lippelt et al., 2014), there is increasing evidence that musical training enhances both untrained but related skills and entirely unrelated skills—which amounts to near and far transfer in cognitive-training terms (Li et al., 2008), respectively. In the following, we will review the available behavioral and neuroscientific studies on these kinds of effects and assess the degree to which musical exercise generalizes to cognitive functions not directly related or unrelated to musical abilities in healthy humans.

## Neuroplasticity

Jäncke (2009) suggested that learning to play an instrument induces structural and functional changes in the brain, due to the continuous activation of the brain regions underlying or engaged by this learning processes. Of particular interest for our purposes, differences between musician and non-musicians might be visible not only in brain areas related to the processing of visual and auditory information but also in frontal regions related to higher-order cognitive control processes involved in producing music. Using voxel-based morphometry, researchers have analyzed gray matter density of people that varied with respect to the intensity of musical training (James et al., 2014). Greater musical expertise was associated with increased gray matter density in the left inferior frontal gyrus, which is involved in syntactic processing, executive functions, and working memory, and in the left intraparietal sulcus responsible for visuomotor coordination. Gray matter density was also significantly increased in brain areas involved in visual pattern recognition (right fusiform gyrus) and in tonal sensitivity (right mid orbital gyrus).

Previous studies have reported musician-enhanced neuroplasticity effects not just during childhood (e.g., Hyde et al., 2009a,b), which is typically much more sensitive to plastic changes, but also across the life span. For instance, a very recent school-based longitudinal study has provided evidence that in-school musical training can affect developmental plasticity even when it is begun in adolescence (Tierney et al., 2015) – a finding that suggests that adolescent brain is still receptive to training notwithstanding the fact that childhood-associated plasticity has started to decline (Penhune, 2011). Another very recent study has shown that such an association between musical training and neuroplastic benefits extends to older individuals for whom plasticity is even weaker (Bidelman and Alain, 2015). Specifically, it was found that, compared to older adults with little-to-no musical training experience, older musicians showed increased neuroplasticity in auditory stem and cortex. This finding is particularly intriguing as it suggests that musical training can have long-lasting effects to the extent that life-long musical engagement can potentially compensate for neuroplastic age-related declines in auditory brain processing associated with normal aging. This implies that life-long musical training may provide musicians with a cognitive reserve that could delay or even reverse age-related cognitive declines (Alain et al., 2014). However, more research is needed to verify musical training impact on the aged brain and to provide evidence for a causal relationship between musical experience and neuroplasticity in the elderly.

## Phonemic Awareness, Reading Abilities, and Language

As pointed out in the introduction, it is not surprising that engaging in learning a music instrument is related to an enhancement of music related abilities. For instance, 2 years in-school music training was found to improve the neurophysiological discrimination of similar speech sounds in children at high risk for learning problems (Kraus et al., 2014). Similar studies have shown that children who underwent years of musical training excelled in the discrimination of small pitch variations in melodies (Magne et al., 2006; Moreno et al., 2009). Neurophysiological studies of the same sort have shown that this skill is accompanied by an increase of error-related negativity in the event-related-potential (ERP) when listening to incongruent tones as well as shorter latencies in positive ERP components (Magne et al., 2006; Moreno et al., 2009). Interestingly, this enhancement in the discrimination of pitch variation in melodies was associated with better performance in the discrimination of small pitch variations in spoken sentences as well—an indication of at least medium transfer of musical training. It is possible that this observation is related to the finding that people suffering from dyslexia have more deficits in a pitch-discrimination-task when they have to find variations in frequency in normal tones than controls (Baldeweg et al., 1999; Besson et al., 2007). Moreover, in preschoolers there seems to be a link between phonological awareness, the ability to conceive the tone structure of heard words, musical skills and reading abilities (Anvari et al., 2002): more musical abilities was positively correlated with better reading abilities and phonemic awareness. All these findings suggest that phonological awareness and music perception may share the same underlying structural mechanisms. Additional evidence supporting the possibility that musical training may have a positive effect on linguistic abilities comes from two recent longitudinal studies in which 8- to 10-year-old children underwent a 2-years music or painting training program (François et al., 2013; Chobert et al., 2014). Compared to children who were assigned to the painting training, those assigned to the music training showed improved speech segmentation skills (François et al., 2013) and enhanced preattentive processing of syllabic duration and voice onset time already after 12 months of training (Chobert et al., 2014). Along the same lines, Slater et al. (2015) reported the first longitudinal evidence that speech-in-noise perception improves after 2 years of group music training.

The influential shared syntactic integration resource hypothesis by Patel (2003) has postulated shared processing structure in language and music, which may account for the observed transfer effects (for an extensive discussion, see Moreno and Bidelman, 2013, and White et al., 2013). Evidence favoring this possibility was recently provided by the study of Moreno et al. (2015), in which 4- to -6-year-old children underwent to a 4-weeks of either a language or a music training. At the post-training evaluation, both groups showed improved processing of trained sounds, which was associated with a better ability to suppress irrelevant (untrained) sounds. Importantly, identical (although attenuated) training-related effects were observed at a follow-up evaluation carried out 1-year after the training intervention.

Very recently Slevc and Okada (2015) have suggested that one shared resource of language and music may be the prefrontal cortex, which is known to be involved in the detection and resolution of conflicts and violations of expectations (van Veen and Carter, 2006). As musical processing encompasses the online processing and integration of musical elements together with the generation of musical predictions and expectations, musical training may well strengthen the ability to detect and deal with conflict.

## Executive Functions, Verbal Memory, and Visual Attention

Making music with an instrument requires several skills involving executive functions: notes have to be played in the correct sequence, with the correct duration and the right temporal distance between them. Indeed, musicians outperformed non-musicians in conflict monitoring tasks suggesting that extended musical experience enhances executive control (Bialystok and DePape, 2009). Even though a recent study has found no evidence of enhanced visual memory ability in musicians compared to non-musicians (Rodrigues et al., 2013), long-term musical training has been found to be related to improvements in working memory (George and Coch, 2011) in both auditory and visual domains and in terms of both behavioral (faster WM updating) and ERP measures (larger P300 amplitude). Along the same lines, musicians outperformed non-musicians in working memory for musical sounds due to an enhanced ability to exert sustained cognitive control (Pallesen et al., 2010). Consistent with this picture, adult musicians compared to non-musicians showed enhanced performance on measures of cognitive flexibility and working memory (Zuk et al., 2014), again supporting the hypothesis that musical training facilitates the development and maintenance of certain executive functions. In a seminal longitudinal study by Bergman Nutley et al. (2014) participants’ performance was evaluated at two/three time points, 2 years apart. It was found that musical practice was positively associated with both visuo-spatial and verbal WM capacity across all three time points. Interestingly, fluctuations in WM between the time points was proportional to the weekly hours spent on musical practice.

Several studies investigated whether musical training improves verbal memory. In comparison to non-musicians, musicians remembered more words from a recently presented word-list (Chan et al., 1998). This finding is consistent with a study in which musicians showed superior performance in verbal memory and reasoning in comparison to non-musicians (Brandler and Rammsayer, 2003). Verbal memory span seems also enhanced with musical training supporting the idea of a superior verbal rehearsal mechanisms in musicians (Franklin et al., 2008).

Visual attention also seems to be improved in musicians (Rodrigues et al., 2014). This finding was confirmed by a recent longitudinal study in which musical training was found to increase visual attention over time (Roden et al., 2014). Very recently, Martens et al. (2015) investigated musical training on the allocation of attention over time as indexed by the “attentional-blink (AB)” —when two target stimuli (T1 and T2) embedded in a rapid stream of events are presented in close temporal proximity, the second target stimulus is often not noticed. People who were musically trained showed an attenuated and delayed AB when required to identify two auditory targets amongst a stream of non-targets, probably reflecting a more efficient allocation of attention. However, this effect was restricted to the auditory domain and did not transfer to the visual domain suggesting a domain-specific effect.

## Processing Speed, Intelligence, and Creativity

Information processing speed is an important mental ability for learning. Musical training seems to have a beneficial effect on processing speed: adolescents with years of active musical experience showed better performance in both visual and auditory information processing speed tasks than non-musicians (Bugos and Mostafa, 2011). These findings may suggest that musical training influences the integration of visual and acoustic information with complex motor patterns. Consistent with this picture a longitudinal study has shown better performance in processing speed tasks in a group of children who followed musical training for 1 year compared to children who followed a science training (Roden et al., 2014). Additional evidence comes from the already earlier mentioned study by Bergman Nutley et al. (2014), in which music training, besides improving WM, was also found to improve processing speed and reasoning ability (i.e., fluid intelligence). These findings are particularly intriguing given the relation between WM and processing speed (Fry and Hale, 2000), and the strong connection of this relationship with the construct of intelligence. The possibility that reasoning ability may benefit from music training is indeed supported by other findings. After 30 weeks children receiving music instruction improved in the Binet intelligence subscale involving spatial-temporal reasoning abilities (Bilhartz et al., 1999). Along the same lines, Schellenberg (2004) found that music lessons, compared to drama lessons or no lessons, enhance IQ, as measured by WISC-III (Wechsler, 1991). Schellenberg (2006) reported a long-lasting association between formal exposure to music in childhood and both IQ and academic performance. Moreover, Moreno et al. (2011) pointed out that even a shorter-term music training of 20 days was enough to enhance verbal intelligence in children. Finally, Schellenberg (2011) suggested that the link between musical training and IQ may not be mediated by executive functions but children with higher IQs may be more likely than children with lower IQs to take music lessons.

Finally, a near-infrared spectroscopy study has shown musical experience to be associated with greater bilateral frontal activity in musicians, compared with non-musicians, during divergent thinking (Gibson et al., 2009). However, more recently, Woodward and Sikes (2015) challenged the notion that musical training enhances creativity in general. They found that musicians scored significantly higher on general creativity assessments than non-musicians only when the tests involved the use of sound stimuli to elicit original responses. In contrast, no significant differences between the two groups when the general creativity assessments involved the use of words and imagery.

## Conclusion

All in all, our review points to an association of musical training and enhanced cognitive performance spanning from executive functions to creativity. That is, even if in some cases the benefits were restricted to the auditory domain, the available studies suggest that musical experience is linked to benefits in unpracticed tasks and cognitive functions unrelated (or at least not obviously related) to musical abilities. However, it is important to note that the causal relation between the observed benefits and musical experience may not be straightforward, although the results of the reviewed longitudinal studies are encouraging. Furthermore, as pointed out by Schubert and Strobach (2012), in training studies in general, expectation effects may contribute to a possible confound in interpreting the results because the knowledge of a study’s hypothesis may change subjects’ behavior in the study. Second, it would be advisable to employ large test batteries which enable the interpretation of the results in terms of level of cognitive processes and not individual tasks (Green et al., 2014). Additionally, the selection of the proper control group is a relevant issue in musical training. Indeed, active control groups are required given that simple test–retest or passive control group do not rule out potential confounds that do not permit a meaningful interpretation of the results. As pointed out by Schellenberg (2005), ideally, the active control should be another activity from the arts, such as drama training. The good thing of such an active control is that it is typically adaptive by nature (i.e., theater lessons tend to increase in difficulty according to the progression of the acting course). Moreover, it is not to exclude that personality (Corrigall et al., 2013) and preexisting neuro-developmental factors may play a mediating role. For instance, individuals with a genetic predisposition that favors executive control functions might be drawn to music more strongly, so that what looks like an effect of practice might actually represent a kind of self-selection. Addressing this issue will require (more) longitudinal studies (Bergman Nutley et al., 2014; Roden et al., 2014). Finally, similarly to other fields of cognitive training (Colzato et al., 2014), it may be interesting to consider individual differences more systematically. Indeed, if musical training really affects neuroplasticity, it makes sense to assume that the effect of music on performance depends on the pre-experimental performance level of the individual—be it in terms of compensation (so that worse performers benefit more) or predisposition (so that some are more sensitive to music interventions).

In sum, our review sheds light on the potential association of musical training for optimizing cognition in general. Similarly to video game practice (Bavelier et al., 2012) and meditation (Lippelt et al., 2014), musical engagement may be a useful cognitive training to promote cognitive enhancement in inexpensive, efficient, and healthy ways—perhaps even for those not genuinely interested in music. In particular, musical training may be a very valuable approach to counteract the deteriorative effect of aging on cognitive functioning (Bidelman and Alain, 2015).

## Author Contributions

Author LC and SB developed the concept and wrote the first draft of the manuscript. Author RS managed the literature searches. All authors contributed to and have approved the final manuscript. approved the final manuscript.

## Conflict of Interest Statement

The authors declare that the research was conducted in the absence of any commercial or financial relationships that could be construed as a potential conflict of interest.

The reviewer and handling Editor declared their shared affiliation, and the handling Editor states that the process nevertheless met the standards of a fair and objective review.
